# Evaluation of a rapid diagnostic test, NanoSign^® ^Influenza A/B Antigen, for detection of the 2009 pandemic influenza A/H1N1 viruses

**DOI:** 10.1186/1743-422X-7-244

**Published:** 2010-09-20

**Authors:** Gyu-Cheol Lee, Eun-Sung Jeon, Won-Shik Kim, Dung Tien Le, Jong-Ha Yoo, Chom-Kyu Chong

**Affiliations:** 1 Water Analysis and Research Center, K-water, Daejeon 306-711, Republic of Korea; 2 Department of Biochemistry, Division of Life Science, Chungbuk National University, Cheongju, Chungbuk 361-763, Republic of Korea; 3 College of Pharmacy, Chungbuk National University, Chungbuk 361-763, Republic of Korea; 4 Research Team for Vectorborne Diseases, National Agriculture Research Center, Kannondai 3-1-1, Tsukuba, Ibaraki 305-8517, Japan; 5 Department of Laboratory Medicine, National Health Insurance Corporation Ilsan Hospital, Goyang 410-719, Republic of Korea

## Abstract

**Background:**

This study evaluated the clinical accuracy and analytical sensitivity of the NanoSign^® ^Influenza A/B antigen kit in detecting 2009 pandemic influenza A/H1N1 viruses. The kit is one of the most popular rapid diagnostic tests for detecting influenza in Republic of Korea.

**Results:**

The NanoSign^® ^Influenza A/B kit resulted in 79.4% sensitivity and 97.2% specificity compared to RT-PCR in the detection of the viruses from 1,023 specimens. In addition, the kit was able to detect two strains of novel influenza viruses, Influenza A/California/12/2009(H1N1) and clinically isolated wild-type novel influenza A/H1N1, both of which are spreading epidemically throughout the world. In addition, the correlation between NanoSign^® ^Influenza A/B test and conventional RT-PCR was approximately 94%, indicating a high concordance rate. Analytical sensitivity of the kit was approximately 73 ± 3.65 ng/mL of the purified viral proteins and 1.13 ± 0.11 hemagglutination units for the cultured virus.

**Conclusions:**

As the NanoSign^® ^Influenza A/B kit showed relatively high sensitivity and specificity and the good correlation with RT-PCR, it will be very useful in the early control of influenza infection and in helping physicians in making early treatment decisions.

## Background

The novel influenza A/H1N1 virus has spread to most of the world's populations, and its spread has led to a pandemic alert situation [1-3]. As a result, at the end of 2009, the World Health Organization announced that the novel influenza A/H1N1 had reached pandemic status [4].

A variety of different diagnostic methods can be used to detect the presence of influenza viruses in respiratory specimens such as nasopharyngeal aspirates, including direct antigen detection tests, virus isolation in cell cultures, and detection of influenza-specific RNA by real-time reverse transcriptase (RT)-polymerase chain reaction (PCR) [5-10]. Albeit the gold standard for the diagnosis of influenza is virus isolation using chicken embryos or tissue culture method, it has the shortcomings such as time consuming and labor intensiveness; it takes between two to 14 days before results are available. Detection of virus-infected cells in nasopharyngeal secretions by direct or indirect immunofluorescent staining is widely used, but it is a difficult and technician-dependent technique still requiring two hours to complete [11].

For the effective control and treatment of novel influenza, rapid and cost-effective diagnosis is important. Rapid diagnosis of influenza allows the physician to begin antiviral treatment, thereby helping control nosocomial transmission of the infection [12,13]. It also helps reducing costs and hospital stay. Rapid diagnostic tests (RDT), known as lateral flow rapid tests, have previously been shown to be cost-effective in pediatric patients [14,15] and effective in controlling influenza epidemics in geriatric institutions [12,16].

The NanoSign^® ^Influenza A/B test is a rapid diagnostic test, which detects the viral nucleoprotein antigen of influenza virus. This kit has been popularly used in Korea and by Korean governmental organizations, including Korea Centers for disease control and prevention (CDC), since its high accuracy has been demonstrated, with a high sensitivity and specificity against seasonal influenza A viruses, including A/H3N2, A/H1N1 (seasonal) and H2N2 [17,18].

This study evaluated the clinical accuracy and analytical sensitivity of the NanoSign^® ^Influenza A/B kit in detecting novel influenza A/H1N1. Using two types of novel influenza A/H1N1, A/California/12/2009(H1N1) and clinically isolated wild type influenza A/H1N1, the sensitivity and detection limits of the NanoSign^® ^Influenza A/B kit were evaluated in this study.

## Results and Discussion

### Clinical data

A total of 1,023 specimens were tested in this study. Among the samples, 199 cases were confirmed to be positive against novel influenza by conventional RT-PCR assay. All specimens were subjected to NanoSign^® ^Influenza A/B test. As shown in Table 1, the kit resulted in 79.4% sensitivity and 97.2% specificity (*p *< 0.001). Sensitivity was calculated by the number of positives recognized by the kit divided by the number of positives identified by RT-PCR assays, and expressed as a percentage [19]. Similarly, the specificity was calculated by the number of negatives recognized by the kit divided by the number of negatives identified by RT-PCR assays, and expressed as a percentage [19]. Table 2 showed a more detailed presentation of the results for novel influenza virus by institution, with either results of NanoSign^® ^Influenza A/B antigen kit or conventional RT-PCR. Positive results seemed to be more frequently observed in general hospitals than local ear-nose-and-throat (ENT) clinics. This can be attributable to the current Korean Flu Prevention & Control System. A person with flu-like syndrome first admits a local ENT clinic near home and if it turns out to be influenza infection than the patient is referred to the general hospital for confirming whether it is novel influenza A/H1N1 infection or not. Thus, a higher rate of positive tests for novel influenza virus is an expected finding.

**Table 1:** Performance of NanoSign^® ^Influenza A/B Antigen kit for the detection of novel influenza A/H1N1

		Results of conventional RT-PCR		Total
			
		Positive	Negative	
Results of NanoSign^® ^Influenza A/B antigen kit	Positive	158	23	181
	Negative	41	801	842

Total		199	824	1,023

**Table 2:** Detailed results for the detection of novel influenza viruses by institutions

Hospitals	Results of NanoSign^® ^Influenza A/B antigen kit/Results of conventional RT-PCR	
Ilsan Hospital (Ilsan city), n = 170	93/120	48/50

Chungbuk National University Hospital (Cheongju city), n = 123	18/23	96/100

Local ENT Clinics (Korean domestic), n = 730	47/56	657/674

Total, n = 1,023	158/199	801/824

Values obtained by the NanoSign^® ^Influenza A/B test showed a considerably high accuracy in the detection of novel influenza. Some studies have shown a very low sensitivity (about 50%) using other RDT kits that are predominantly used in the market, even though the kits have a high accuracy for the detection of seasonal influenza viruses [20,21]. In addition, the correlation between NanoSign^® ^Influenza A/B test and conventional RT-PCR was approximately 94%, indicating a high concordance rate.

### Analytical sensitivity

**Figure 1:**
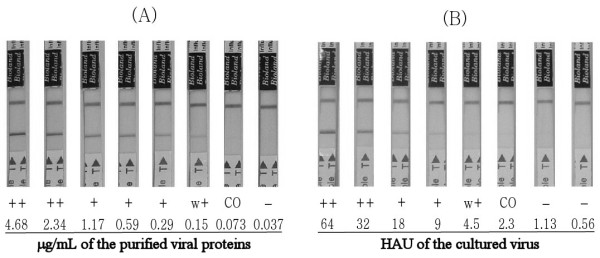
**Analytical sensitivity of NanoSign^® ^Influenza A/B for novel influenza viruses**. (A) The novel influenza A/California/12/2009(H1N1) viruses were cultured in the fertilized chicken embryo, harvested from the allantoic fluid, and purified to homogeneity using density gradient ultracentrifugation (3% sucrose), as described [26]. The purified viruses were serially diluted in 2-fold manner and tested with the RDT kit. Bradford protein assay was used for the protein quantification. (B) The clinically isolated wild type novel influenza viruses were cultured in MDCK cell line. When the cytopathic effect was observed, the cultured viruses were harvested. The viral stocks were serially diluted in 2-fold manner and tested with the RDT kit. Hemagglutination assay was used for the titration of the viruses. w+, weak positive; CO, cut-off value.

To date, there are few studies on the analytical sensitivity of commercialized RDT kits for detecting novel influenza viruses [9,22,23]. In this study, chicken embryos were used to culture the novel influenza A/California/12/2009(H1N1) and then the cultured viruses were purified to homogeneity using density gradient ultracentrifugation (Figure 1). Using the pure virus solution, the analytical sensitivity of NanoSign^® ^Influenza A/B was 73 ng/mL of the purified novel influenza viral proteins (multiple testing revealed 5% deviation, ± 3.65 ng/mL) (Figure 1A). In addition, in the case of the wild type novel influenza virus found in Korea, the sensitivity was 1.13 ± 0.11 hemagglutination (HA) unit with possibly about 10% deviation (Figure 1B). Several wild types of novel influenza viruses have been tested for the detection limit of the kit and all cut-off values were within the range of 1 to 3 HA units.

Rapid influenza diagnostic tests are antigen-detection tests that target the nucleoprotein of the virus. Usually, the commercially available RDT kits provide results within less than 30 minutes. Thus, results are available in a clinically relevant time frame to help assist in the clinical decision-making process, thereby preventing secondary infection. Some studies have reported that RDT kits have a low to moderate sensitivity against novel influenza compared to RT-PCR [24]. However, the analytical sensitivity of RDT kits is dependent on the properties of its antibody. According to this clinical evaluation of the NanoSign^® ^Influenza A/B antigen kit, its sensitivity for novel influenza (79.4%) was significantly higher than commercialized detection kits (10-70%) that have been reported to the CDC [25]. There are many factors that can affect the sensitivity of the RDT kit, since, unlike RT-PCR, it does not have any target amplification step. Factors that contribute to the sensitivity of the RDT include technician skill in collecting specimens, the type of specimen, the quality of the specimen, the stage of illness when the specimen was collected, and patient age. In addition, the physician's ability to obtain a good specimen is a key factor in obtaining higher sensitivity.

RDT kits such as the NanoSign^® ^Influenza A/B antigen kit are very useful in the early control of influenza infection and in helping physicians in making early treatment decisions. However, the negative results of this particular RDT kit used in this study do not rule out influenza virus infection because its sensitivity is not 100%. For this reason, when there is a high clinical suspicion of influenza infection in a patient, empirical antiviral therapy should be administered. Finally, when reporting the results of a RDT kit, it should be better to inform the physicians about the limitations of the test, so that they can decide the most effective clinical approach.

## Conclusions

The NanoSign^® ^Influenza A/B kit had 79.4% sensitivity and 97.2% specificity in the detection of the viruses, and could detect two strains of novel influenza viruses, Influenza A/California/12/2009(H1N1) and wild-type novel influenza A/H1N1. Analytical sensitivity of the kit was approximately 73 ± 3.65 ng/mL for the purified viral proteins and 1.13 ± 0.11 HA units for the cultured virus. In addition, the correlation between the NanoSign^® ^Influenza A/B test and conventional RT-PCR was 94%, indicating a high concordance rate. The NanoSign^® ^Influenza A/B kit may be very useful in the early control of influenza infection and in helping physicians in making early treatment decisions.

## Methods

### Viruses

Influenza A/California/12/2009(H1N1) was kindly provided by the Laboratory of Virology, Chungbuk National University and wild-type novel influenza A/H1N1 was obtained from the Department of Laboratory Medicine, National Health Insurance Corporation Ilsan Hospital.

### A host cell and chicken embryos

A host cell, MDCK, for the culture of the viruses from nasal swabs and aspirates was kindly provided by the laboratory of Professor Chan-Hee Lee. Chicken embryos were purchased from an egg store in Korea (Ochang Mart, Ochang, Republic of Korea).

### Virus culture

For the culture of Influenza from the aspirates of nasal swabs, MDCK cells were cultured in DMEM (Invitrogen Corporation, Carlsbad, CA) supplemented with 5% fetal bovine serum (FBS, Invitrogen Corporation) at 37°C and 5% CO_2_. Before infection, MDCK cells were trypsinized in 1 × 10^7 ^cells per T75 flasks (Nalge Nunc International, Rochester, NY). After seeding in 10 mL DMEM plus 5% FBS at 80-90% confluency, plated cells were rinsed with phosphate-buffered saline (PBS, Invitrogen Corporation). Suspended specimens in the flasks were gently shaken for two hours for effective mixing. Cell infections were observed until 80-90% of the cells were floating or lightly attached to the T75 flask (typically 4-5 days post-infection). At that time, harvesting and storage of viral supernatant was performed.

### Hemagglutination assay

The titer of the influenza A/H1N1 virus was determined by HA assay. First, U-bottom 96 well plates (Nalge Nunc International) were prepared and 50 μL of PBS (pH7.0) was added to each well. Next, after adding 50 μL of original viral solution, they underwent two folds serial dilutions. Finally, 50 μL of 0.5% turkey red blood cells were added and mixed. The mixture was then incubated for approximately 30 minutes at room temperature. The number of positive reactions showing agglutination was observed and recorded to calculate virus titer.

### Immunochromatography assay

To prepare the nasal swab specimens, a sterile swab was carefully inserted into the nostril with the most secretions under visual inspection. Using a gentle rotation, the swab was pushed until a weak resistance occurred at the level of the turbinate (less than one inch into the nostril). Next, the swab was rotated a few times against the nasal wall. To test the RDT kit, a test strip was inserted into the tube containing the 300 μL of extraction solution and allowed to sit at room temperature prior to testing. After preparing the nasal swab, the sample was inserted into the tube and swirled at least six times while pressing the head against the bottom and side of the tube (the swab head should be rolled and squeezed against the inside of the tube as it is removed). Finally, the test strip was inserted into the tube containing the sample-extracted solution. The results were interpreted after 10 minutes. Some positive results appeared sooner, but results were not read after 30 minutes.

### Conventional RT-PCR

For confirmation of cultured influenza A/H1N1, the viral RNA was isolated from the infected MDCK cells. RT-PCR was performed with the commercialized Influenza A(H1N1) Detection Kit by using primers targeting the novel influenza (GeNet Bio, Nonsan, Republic of Korea). RNA was directly extracted from the specimens or virus cultured-supernatant using the QIAGEN^® ^Viral RNA mini kit (Qiagen, Hilden, Germany). cDNA synthesis was accomplished at 42°C for 30 minutes. Next, the DNA was amplified by 40 cycles of PCR with three steps: denaturation at 94°C for 20 seconds, annealing at 54°C for 20 seconds, and elongation at 72°C for 30 seconds. Finally, an additional elongation step (72°C/5 min) was carried out. The amplified gene products were analyzed with 2% agarose gel electrophoresis. The size of the amplified target was 170 base pair for novel influenza A/H1N1 and 350 base pair for seasonal influenza A/H1N1.

## Competing interests

The authors declare that they have no competing interests.

## Authors' contributions

CKC and GCL conceived this study. GCL, ESJ, CKC designed the experiments. ESJ and DTL cultured cells and viruses. GCL and ESJ carried out the RDT and conventional RT-PCR. WSK and JHY collected specimens in clinics and carried out RDT. GCL, WSK and CKC analyzed the data. GCL and CKC wrote the manuscript. All authors read and approved the final manuscript.
